# The morality and tax avoidance: A sentiment and position taking analysis

**DOI:** 10.1371/journal.pone.0287327

**Published:** 2023-07-17

**Authors:** Mark Lokanan

**Affiliations:** Faculty of Management, Royal Roads University, Victoria, BC, Canada; Yunnan Technology and Business University, CHINA

## Abstract

This paper examines the moral and legal underpinnings of corporate tax avoidance. Cast in terms of a totemic symbol that brand tax avoidance as within the purview of the law, the paper invokes the attributional frames of the new sociology of morality to examine the position of both the moral advocates and the amoral critics of aggressive tax avoidance. The paper uses the United Kingdom as a jurisdiction where complex tax planning by tax advisors serves as a measure of protection for corporations who may have already conceived that they are paying too much tax. Data for the paper came from semi-structured interviews conducted with tax accountants, consultants, parliamentarians, and government officials. To supplement the interviews, data from the Parliamentary Commission on Banking Standards were collected and analyzed to provide useful insights. The findings reveal that through effective tax planning, companies can reduce the present values of future tax payments. Given the singular justification of their actions within the contours of the tax rules, the moral culpability of organized tax avoidance is minimized, with very little liability attached. Tax avoidance is a morally charged area that is slowly drifting away from conventional social norms of what is right or wrong. It is hard not to see those in charge of tax regulation not using the findings of this paper to provide a more nuanced understanding of the intractable problems associated with corporate tax avoidance and use it as a reference point for regulatory reforms.

## 1. Introduction

In recent years, the United Kingdom (U.K.) has experienced a spate of tax avoidance cases involving some of the world’s largest conglomerates. Global and local firms such as Google, Amazon, Starbucks, Vodafone, Next, and, more recently, Cargill and Goldman Sachs, were all implicated in one way or another in tax avoidance schemes. These companies all have highly complex tax structures, but a recent spate of stories highlighted in the press has made tax avoidance a topical issue [1–3]. In 2012, for example, Starbuck had sales of £400m in the U.K., but because of aggressive tax planning, the company paid no corporate tax [4]. It is alleged that Starbucks was able to reduce its taxable income in the U.K. by regularly paying a royalty fee to the company’s headquarters in Amsterdam; as such, it faced a much lower rate of taxation relative to the U.K. [5].

Meanwhile, the U.K. parliamentary committee has grilled Google twice over its tax practices. Google had a U.K. tax bill of £35 million pounds in 2012 on sales of £4.9 billion to British customers [6]. It is alleged that Google ‘paid a tax rate of 2.6 percent on £5.1 billion in non-U.S. income in 2012, because it channelled almost all of its overseas profits to a subsidiary in Bermuda, which levies no corporate income tax’ (para. 2). Likewise, Amazon’s British business paid only £4.2m in tax last year, despite selling goods worth £4.3bn [7]. More recently, ‘US titans Cargill and Goldman Sachs have been found guilty of tax avoidance and subsequently handed a £79m (€89m) bill’ [8]. Added to these cases, the number of individuals charged and prosecuted for tax-related crimes has halved in the past five years despite political pressure by the U.K. tax authority to tackle the billions of pounds lost to tax fraud every year [9].

Under public pressure, the legislative body in the U.K. commissioned the Public Accounts Committee (PAC) to investigate aggressive tax avoidance by multinational corporations. During one of the hearings, the chair of the PAC, Margaret Hodge, hinted on the moral valuations of corporate tax dodging with the following comments: ‘People have invested in your scheme to take advantage of the allowances, so they avoid tax. You may say that is legal; I think it’s immoral… It’s the old argument’ [10]. As with any tax avoidance schemes, the systematic fault lines and moral dimensions must be probed to expose the sordid culture surrounding the nature of organised tax avoidance.

Adopting this analytical lens provides ideal fodder for a sociological analysis of the actions of moral agents to contrive complex tax avoidance schemes. In this article, the new sociology of morality is invoked as a frame of reference to examine the position of both the moral advocates and amoral critics of aggressive tax avoidance. The objective of this paper is to conduct a sociology of morality analysis to explore how corporate tax avoidance is represented by various stakeholders in the U.K. The following research question guides the inquiry: How do the different groups (e.g., tax accountants, politicians, tax consultants, professors) differ in their understanding and representation of what is moral or immoral tax avoidance?

Against a changing professional context, an attempt is made to understand the sentiments of different stakeholders on the issue of tax avoidance in the U.K. Sentiment analysis is a technique used to process natural language and analyze the emotional tone of texts. We use sentiment analysis to analyze textual data obtained from interviews and witnesses’ testimonies and understand the emotional tone of their responses [11]. More specifically, the natural language processing (NLP) component is integrated into the research design to extract more granularities from the data and compare the tones of participants with the actual transcripts. NLP first identifies the important topics and then sorts them in order of importance. The themes identified by the NLP technique are then compared with the interviews and witnesses’ testimonies.

We use the U.K. as a jurisdiction where complex tax planning (a euphemism for tax avoidance and evasion) protects corporations that may have already conceived that they are paying too much tax. Cast in terms of judicious tax planning, this sort of moral minimalism by corporations represents a situational morality that neutralizes the reducing tax effect as the natural order of normal economic transactions. This paper significantly contributes to the tax avoidance literature in addressing these issues.

### 1.1. Contributions

Firstly, the sociology of morality is an umbrella term that covers a wide range of topics related to morality and social behaviour. While the sociology of morality has been widely studied in academic journals, it has yet to be applied to the real world as much as it could be. This paper seeks to change that by using the sociology of morality to understand tax avoidance. Tax avoidance is a moral issue because it involves breaking the law to gain a financial advantage. By understanding the social factors that contribute to tax avoidance, we can begin to address the problem at its root. The sociology of morality can help us to understand why people feel justified in breaking the law and how we can create a society that discourages tax avoidance.

Secondly, the widespread practice of tax avoidance sits in stark contrast to the moral rhetoric of corporate codes of conduct and their pronouncements about the need to be good corporate citizens [12]. Consistent with prevailing corporate cultures that shape individuals to fit organizational environments [13], professional tax advisors’ actions to minimize their clients’ tax liability reflect moral reasoning that cannot fairly justify their actions. A new sociology of morality is emerging, cloaked in manipulation and deception and now seen as the normal feature of structured capital markets. The goal is to unpack this attributional framing of morality and fairness to provide insights into the seedy underbelly of organized tax avoidance. This perspective offers a unique contribution to understanding the repositioning of the relationship between tax avoidance that is considered to be within the ambit of the law, and that is seen as violating the law. The recognition of this unique epistemology opens up a window of opportunity for regulators and standard setters to view and apply strategies to address organized tax avoidance.

The rest of this paper is structured as follows. Section 2 discusses the literature on the new sociology of morality concerning corporate tax avoidance, followed by a discussion on the role of emotions in sociological settings. Section 3 describes the research design, data sources, and analytical techniques. Section 4 presents an analysis of the findings, while Section 5 presents the sentiment and emotional analysis. Section 6 presents the conclusion and highlights the policy implications.

## 2. Literature review

### 2.1. The basic assumption of the new sociology of morality

The New Sociology of Morality is an area of sociology that considers how morality has changed and is changing in modern society [14]. It takes a particular interest in the ways that new technologies, social media, and changes in the laws are affecting morality. The New Sociology of Morality is not concerned with whether something is right or wrong but rather with how people decide what is right or wrong [15–17]. This makes it different from classical sociology, which tends to take a more value-neutral approach to social interaction. The New Sociology of Morality is still a relatively new area of study, but it has already significantly impacted our understanding of morality [14,18–22].

Ever since the global financial crisis of 2007–2008, there has been a resurgence in the New Sociology of Morality to explain the unchecked greed of corporate officials [12,14,17,23,24]. The new sociology of morality, as understood from this scholarship, emphasises the role morality plays to ‘bind people together through a common system of rules and expectations during interactions’ [22]. The development of the new sociology of morality has left no space for grand ideas about moralistic progression, evolutionism, and structural functionalism, which were all once the hallmark of classical moral sociology [18]. Framed with reference to the moral order and market society, the new sociology of morality explains the tense relationship among the nature, causes, and consequences of people’s actions and their ideas about what is good and bad in the moral order [25]. The new sociology of morality does not use terms such “culture”, “values”, and “norms” as a normative to explain social phenomena, but rather as a “cognitive ‘toolkit’ or ‘repertoire’ made up of ‘rule-like structures’ that serve as ‘resources that can be put to strategic use’ [26]. The focus is more on understanding the role that cultural normativity plays in understanding cognitive moral judgments (i.e., understanding the shift from normative to cognitive) [15,17–19,26].

The New Sociology of Morality has challenged the idea that there is one morality that is fixed and unchanging [16–18,26]. This is known as moral relativism [27,28]. The New Sociology of Morality posits that morality is always changing, and that what is considered right or wrong can vary from place to place and from time to time [18,29]. This means that there is no such thing as an objective morality, and that what is considered moral depends on context and the human interaction [14,20,25]. The New Sociology of Morality also challenges the idea that morality is something that is passed down from generation to generation. Morality is constantly evolving, and that new generations often have different ideas about what is right and wrong, that is, morality is not something that is static but is always changing [14,25,30]. Considerable efforts have already been made by scholars to integrate the new sociology of morality not so much to explain the interplay between social order and human actions, but to a more substantial integration into the moral causes and movements of social life, boundary works, and valuations processes [20,21,26,31].

### 2.2. Sociology of morality and corporate tax avoidance

This section will discuss corporate tax avoidance through the New Sociology of Morality framework. This line of inquiry means exploring how companies come to evaluate corporate tax avoidance as part of their fiscal strategies. To conduct a moral inquiry of tax avoidance, researchers need to probe deeper to understand how corporations are exposed to a particular context (i.e., the wider economic and regulatory landscape) and develop a moral choice process that is conducive to non-compliance with tax codes [12,32,33]. This outcome requires an understanding of why moral contexts and rules of enforcement emerged and what their roles are in influencing corporations’ moral perceptions relevant to their involvement in tax avoidance schemes. At play in this process is an ethic of normalisation whereby the actions of the corporate techno-structure appear to be wedded to a conception that manipulation and deception are normal features of a well-functioning regulatory system [31,34–36]. The behaviour and decision-making displayed by tax professionals are significantly affected by the value system in which they operate. Working in a complex environment involving issues connected with tax planning and avoidance necessitates a clear understanding of acceptable business practices [37]. Herein lies the problem with corporate tax avoidance. When does a corporation’s avoidance of paying its fair share of taxes become responsible tax avoidance (i.e., perfectly legal within the spirit of the law) and abusive tax avoidance (i.e., against the spirt of the law)? The next section is devoted to providing explanations to these question through the New Sociology of Morality framework.

As is evident by now, an important theme in the tax avoidance literature is the emphasis placed on the *spirit* and *letter* of the law. Like most accounts on tax avoidance, a normative and moral dimension that is referenced and articulated around frames of ‘responsible tax planning’ and ‘good financial governance’ buttress this theme [38]. Some corporations may conceive that avoiding taxes by bending the rules is not illegal because they are operating within the letter of the law and may not have an issue with their conduct [39]. Tax planning aimed to minimized corporate tax liability is valorised as good financial governance [13,33,40]. As the argument goes, avoiding taxes and bending the rules to maximise corporate income is not illegal; these corporations are ‘operating within the letter, but perhaps not the spirit, of the law’ and are basically maximising their values in competitive markets [13,41]. At play in this representation is the notion that companies minimise their tax liabilities by making use of tax professionals at their disposal to gain exemptions and pay lower taxes [35,36].

Then, there is an ethic of normalisation where proponents of legal tax avoidance remain wedded to the conception that corporations are already contributing enough to civic society and should not pay any more taxes. This argument has its roots in the dynamic of rationalisation and is disseminated through a school of thought that has a widespread social aversion to paying corporate taxes [12,36,39]. At play in this representation of fairness is the argument that corporations are already serving the public interest by expending a great deal of their profits on corporate social responsibility activities, and the payment of more taxes would be unfair and fiscally irresponsible [42,43]. Underlying this argument is proponents’ deference to the value of corporations’ social obligations to a wide range of stakeholders, which, when met, fulfil corporations’ moral obligations and should work to minimise their tax burden. The lesson from this discussion so far is that corporations’ social responsibility activities should be considered to mitigate the amount of taxes they pay or to absolve them from paying any taxes [36,44–46]. Although there are compelling arguments for corporations to avoid paying taxes once their actions are within the ambit of the law, there are also persuasive arguments against tax avoidance. These arguments are discussed below.

### 2.3. The moral taints of tax avoidance

The same reference points used to support the arguments for corporate tax avoidance are used to frame arguments against corporate tax avoidance. Framed and mobilised through the lens of accountability and citizenship, the prime social responsibilities of corporations are viewed as paying their fair share of taxes [12,44,47,48]. Corporate activities and their products pervade our lives in many ways [49]. As industrious citizens, we contribute to corporations’ bottom line by purchasing their products; as employees, we assist in maintaining the logistical flow of the corporation; and as professionals, we are put in a position where we have to regulate corporate affairs. In almost every facet, the corporation benefits from the industriousness of the wider public, which by virtue of their intricate involvement become stakeholders in the quality of corporate activities–that is, in both their effectiveness and legality [50]. Given the definitive involvement of the citizenry in contributing to corporate profitability, it is only fair that corporations reciprocate by paying their fair share of taxes to fulfil their societal obligations.

A second complementary argument is that corporations by legal definition are entities of the state with certain privileges granted to them through government regulations [12]. As part of good governance, corporations through their accountants and lawyers,

will seek to minimise their tax liability through ‘tax planning’, making the most of the tools and mechanisms which the government makes available to them specifically for this purpose: allowances, deductions, rebates, exemptions, and so on. Tax planning is tax compliant behaviour but there is a grey area between this and ‘tax avoidance’ [51].

The state, by giving corporations the freedom to structure their operations in such a way as to maximise their profit and shareholders’ value, allows them to generate considerable benefits, with concomitant social impact [52]. As corporations expand their business scope, they must ensure that their records align with established standards, such as the International Financial Reporting Standards (IFRS). These principles guide how these companies should conduct their finances while leaving space for professional judgment. Crucial to the success and longevity of any corporation is the pursuit of profitable endeavours. Naturally, these firms will strive to increase their after-tax profits as much as possible within reasonable limits [35,53,54]. With the government bearing the primary regulatory burden, paying their fair share of taxes is seen as a natural way for corporations to fulfil the obligations the state has granted to them and to satisfy their stakeholders [12,52]. Despite the renaissance of research in the general business literature on the discrete events that lead to organised tax avoidance, very little intellectual attention has been paid to the moral sociology associated with the actions of companies and tax professionals involved in corporate tax dodging. Researchers must take stock of this theoretical stance with its distinctly interdisciplinary character and step outside the ontological box of one-dimensional explanation that has long shaped the discussion of tax avoidance [13,31,38,44,52]. We attempt to address this gap in the tax avoidance literature.

### 2.4. The role of emotional in sociological settings

The role of emotions is central to this paper. For this reason, a brief discussion of the role emotions play in sociological settings is warranted and should be considered [22]. Emotions are intertwined with social phenomena and are a significant part of how we interact with others, make choices, and think about events in a relevant context [17]. They can provide valuable information on what is happening in our environment and may help us understand the social dimensions to which we habituate [55,56]. Emotions are an important aspect of social life [57]. Emotions provide us with information about the social environment while also playing a role in how we interact with others and make choices in various domains [57]. Human-to-human interactions in social contexts are increasingly complex, but there are a few key points to keep in mind [58]. First, emotions are not simply individual reactions to events or experiences. Instead, they are complex social phenomena that are shaped by the centrality of our emotions and interactions with others across various contexts [57]. Second, the inferential process of emotions can have both positive and negative effects on our social lives. Emotions can either help to bind us together (through close relations and group decisions) or drive us apart because of interpersonal strain [55]. Additionally, emotions are incidental and change over time; hence, decisions made based on inconsistent fleeting incidental emotions become difficult to trust [59].

Despite these problems, the role of emotions in social settings remains important and understanding how they work can form the basis of obtaining insight into the semantics of verbal discourse [55,57]. On a basic level, emotions provide information about our environment and can help us identify potential threats or opportunities. Emotions can also motivate us to act and can influence the decisions we make about a particular narrative or action. In social settings, emotions can be used as a form of communication and help create bonds between people and signal interest, approval, or disapproval [59]. As a result, emotions are a crucial part of social interaction, and understanding how they work is essential for understanding the dynamics of social interactions. With respect to emotion, this paper tests the following hypotheses about the relation between emotions and moral judgments:

Hypothesis 1:

**H**_***0***_: There is NO difference in the emotional sentiments and moral judgements expressed by different stakeholders on corporate tax avoidance.
**H**_***1***_: There is a difference in the emotional sentiments and moral judgements expressed by different stakeholders on corporate tax avoidance.
Hypothesis 2:

**H**_***0***_: The level of emotions and moral judgements expressed by stakeholders are NOT affected by the seriousness of the topic.
**H**_*1*_: The level of emotions and moral judgements expressed by stakeholders are affected by the seriousness of the topic.

Equation two represents the formula to calculate the hypotheses.

Where:

p_*1*_-p_*2*_ represents two different sample populations,

with a confidence interval of 95%,

and a critical value of 1.96

$$p_{1}‐p_{2}\pm 1.96X\frac{\sqrt{p1(1-p1)+p2(1-p2)}}{n1+n2}$$ Eq 1

## 3. Methodological approach

To clarify, this paper *conducts a sociology of morality analysis on how various stakeholders in the U*.*K represent tax avoidance*. To answer these questions, 20 individuals were interviewed for this paper. As can be seen from Table 1, the interviewees includes participants from Her Majesty’s Revenue and Customs (HMRC), accountants, tax consultants, politicians, and ethics professors. Textual data in the form of written evidence and transcripts from witnesses’ examinations taken before the Parliamentary Commission on Banking Standards (PCBS) between June 2015 and March 2016, and briefing papers on tax avoidance and evasions in the U.K. were also collected and analysed.

**Table 1:** Interviewees’ background.

Career/Industry	Number of Participants	Average years in industry	Participant Number
HMRC	3	4.3	H1, H2, H3
Tax Consultants	5	5.6	TC1, TC2, TC3, TC4
Accountants	6	5.5	A1, A2, A3, A4, A5, A6
Professors	3	12.6	PR1, PR2, PR3
Politicians	3	4.6	P1, P2, P3
**Total**	20		

### 3.1. Justification of methodological approach

We captured diverse views on tax avoidance using interviews and textual data. Interviewing participants allowed for a more nuanced understanding of tax avoidance in the U.K. The interview data collected was essential for understanding the U.K.’s tax avoidance issue by analyzing the views of individuals who have lived experience in the tax regulation sector. The interview data allowed the research team to probe deeper and collect rich and detailed data on the participants’ experiences, opinions, and attributes on issues related to tax avoidance.

The analysis of textual data on tax avoidance in the U.K. was useful in developing a more comprehensive understanding of this phenomenon beyond just its definition. Textual data were useful for studying and developing insights into the meanings and patterns of language in the written text. The textual data reveal underlying values, beliefs, and assumptions surrounding the culture of tax avoidance by companies in the U.K. In summary, the in-depth interviews and the textual data analysis allow for a more thorough understanding of the research questions and increase the validity and reliability of the findings. The use of in-depth interviews and textual data allowed for the triangulation of the results.

### 3.2. Sampling and representation

According to [60], selecting interviewees who have a direct interest in the issues under investigation provides the best possible results when using interviews. Considering this perspective, coupled with the specific and complex nature of the issues to be investigated in this study, interviewees were chosen from five separate and relevant occupational groups: U.K. governmental tax experts from the HMRC office; business ethics professors with specific knowledge about tax avoidance and the tax profession; tax consultants (i.e., professionals who are educated in law, accounting, business, and finance and specialize in taxation law); career politicians who were and are currently involved in tax legislation; and ACCA and ICAEW qualified accountants who specialized in corporate audits and tax laws and represented both the Big Four and second-tier audit firms. Except for the three ethics professors, Table 1 shows that these respondents have on average about 4 to 6 years of experience to ensure that the participants and context are sufficiently represented in the tax avoidance narratives. The three business ethics professors have researched and written about tax laws and how to avoid them.

Given that we were looking for participants who were closest to the sea of action around tax avoidance issues and had extensive experience in dealing with tax law and tax policies, purposive sampling technique was used to select interviewees. Attempts were made to include professionals who were key informants on issues concerning tax avoidance; who were actively involved in tax reforms and legislation; who were actively involved in tax compliance issues; and who had a wealth of experience in tax crimes. Killian et al. clarify the essential nature of collaboration between tax experts and policymakers when creating effective regulatory frameworks; different perspectives are needed to ascertain the issues faced in various jurisdictions and develop targeted solutions [61]. Traditional regulatory measures may be challenging to implement in the setting of tax havens. However, it is still possible to utilize formal (hard) and informal (soft) mechanisms to promote compliance and boost transparency [31,61].

Purposive sampling technique was employed to attract participants. The first step was to identify prospective interviewees. Once the prospective interviewees agreed to participate in the study, an effort was made to seek out individuals who were known to the prospective interviewees and who were closest to issues related to tax compliance from a national and geo-political perspective. After careful consideration regarding the individual’s expertise, experience, and role in the industry, a decision was made to include them as interviewees. Purposive sampling was effective because it allowed the research team to select participants with diverse experience and roles in dealing with taxation issues.

### 3.3. Data source 1: Interviews

Given the sensitive nature of corporate tax avoidance, it was very difficult at first to find participants who were willing to be a part of the study. Trying to find prospective interviewees was more difficult in the initial stage of the interview process; however, the research team followed the purposive sampling process very closely and was able to conduct semi-structured interviews with individuals that captured insights into some of the more salient issues concerning corporate tax avoidance [62]. Several selection criteria were developed to identify potential participants for this project. The criteria were based on the research objectives and the target population. Only potential participants with the necessary expertise and professional experience to participate in the study were identified. Participants were identified through various mediums, including professional networks, social media, and referrals. We then screened potential interviewees based on these selection criteria and explained the research objectives and purpose of the interviews. If the criteria were met, we scheduled an interview with the participant; if the criteria were not met, we then proceeded to identify participants who met the selection criteria. More important, once the participants had agreed to be interviewed, we had to obtain organisational consent from their employers. Access was difficult to obtain and took months of negotiations. Therefore, for confidentiality and anonymity concerns, the names and entities of participants’ organisations will not be identified.

To capture different views on corporate tax avoidance, individuals who represented different groups and lacked common ground were selected to be a part of the study. The interviews offered a fascinating glimpse into the subterranean cluster of narratives about the nature and attributes of corporate tax avoidance. Although discursive representations of tax avoidance schemes can seemingly be based on the banal lessons learned from cognate regulatory problems, the sheer magnitude of the problems encouraged the interviewees to discuss the undeniable reality of tax cheating as a rational economic behaviour [63]. The final sample had a semblance of diversity and consisted of 20 interviews. To enhance content validity, the interviews were conducted with the aim of reaching data saturation or reaching the point at which no new information or themes could be observed in the data [64,65].

The interviews were conducted over a period of 10 months between 2015 and 2016. Each interview lasted 45 minutes to an hour and consisted of standardised open-ended questions. To aid in the analysis, the interviews were semi-structured, with the interviewees being allowed to speak more freely about tax compliance issues that were neither bound to a specific discursive framework nor beholden to any particular special interest groups or influences [66].

Becker’s [67] work, *Tricks of the Trade*, interview questions were based on the ‘how’ and the ‘what’ rather than the ‘why’ to provide space for interviewees’ expressions and subjective experiences with tax avoidance and compliance. Rather than allowing the interviewees to speak on issues they deemed important, we decided a set of predetermined questions would be asked to all interviewees. These guided questions led to a deeper understanding and a more concentrated discussion of the interviewees’ views and opinions on the morality and legality of corporate tax avoidance. Additional questions were asked at various points to clarify issues or, in the case of expert interviews, to obtain more valuable and detailed insights into the hegemonic construction of tax avoidance schemes. After each interview, a debrief was carried out to review the responses in accordance with the evidence presented and modify the questions where necessary [64,66]. This iterative approach was useful for two purposes. First, it allowed the researcher to fine-tune the research question as more data were collated, and second, it allowed for theory and practice to evolve together with the emergence of new data.

The interviewees were all transcribed in Microsoft Word and uploaded onto NVivo. Each interview was micro-analysed to identify themes to be investigated and explore further. A micro-analysis of the interviews not only assisted in the identification of thematic clusters but also helped uncover general patterns of consensus and critiques in the data. The interview data were categorised and defined in terms of a series of conceptual frames that informed the following thematic and etiological clusters: (1) moral and immoral tax avoidance schemes; (2) gamesmanship of stakeholders involved and affected by tax avoidance schemes; (3) tax avoidance and commercial focused private interest; and (4) moral concerns of tax avoidance. These represented the basic themes on which the participants concentrated when addressing tax avoidance issues. Nodes were created for these themes and coded to their relevant sources in the transcripts.

### 3.4. Data source 2: Accounting firms’ written evidence to the PCBS

To enhance objectivity and validity, the interview data were supplemented with data from the PCBS hearings. Both the U.K. House of Lords and the House of Commons commissioned the PCBS hearings to report on the investigation into the professional standards and culture of the financial services sector and to make recommendations for legislative reforms. The hearings involved witness testimonies in several areas, including morality and ethics in finance; changes in banking culture; corporate governance and operations of wholesale markets; audit and accounting; and tax avoidance and evasion.

The PCBS hearings were conducted between June 2012 and March 2014 and involved 733 witnesses who gave written and oral testimonies to special panels both Houses commissioned. In total, over 40 public hearings were conducted that comprised about 4,418 pages of transcripts with various stakeholders, including former and current parliamentarians, bankers, accounting firms, standard setters, government regulators, and so on. Given the sheer volume of textual data, the selection process was narrowed down to documents that helped answer the specific research questions on the "morality of tax avoidance." The final documents included 2145 pages of testimonies and analyses dealing with morality and corporate tax avoidance issues. The accounting and audit evidence, along with the special panel and hearing on corporate tax avoidance, was of particular interest to this study because they involved senior officials from the big four accounting firms, professional accounting bodies, financial institutions, banks, and the HMRC.

The financial resources available for the PCBS inquiry amounted to about £850,000, allowing the commission to identify suitable experts to conduct a very comprehensive and wide-ranging set of hearings involving numerous stakeholders from the financial sector. The available testimonies and supporting documents were more in-depth than any amount of data a single researcher could have collected to inform their arguments in this paper.

### 3.5. Sentiment analysis

Sentiment analysis using NLP techniques in the Python programming language was conducted on both the interview data and the transcripts from the PSBC. NLP is a deep- learning artificial intelligence (AI) technique used to detect sentiments and emotional values in text and speech [68,69]. Sentiment analysis (a form of text analytics) combines NLP and AI techniques to find meanings in a large corpus of textual data. In this project, sentiment analysis was used to identify the emotional tone of the transcripts of the interviewees and the PCBS inquiries [70,71]. To conduct sentiment analysis, the data were read into Python, extracted on a transcript-by-transcript basis, and collated in a data frame consisting of unique questions along with their respective answers in each row. From the raw file, the data were captured in a list format, and from the list of the overall transcript, the index of questions was identified. Next, all the data obtained from the responses to the consecutive questions were considered and analysed for their sentiments.

Two types of sentiment analysis were conducted: The first assessed the polarity of the sentiments from the interviewees’ responses. The responses were scored as positive, negative, or neutral. The polarity of the data ranged from -1 to +1, with -1 being a completely negative emotion, 0 being neutral, and +1 being a completely positive emotion. The subjectivity of the data ranged from 0 to 1, with 0 being a complete opinion and 1 being a fact [72]. The second sentiment analysis was more granular and assessed the emotional tone of the responses as ‘fear’, ‘sad’, ‘surprise’, ‘angry’, and ‘happy’. Text2emotion was used to code and analyze the data into the five emotions. Text2emotion is a software application that detects emotions—anger, joy, fear, sadness, and surprise—from texts. The app uses NLP algorithms to analyze text data and identify emotion-based patterns. Emotion recognition allows for more granularity of the data and can tease out the emotional responses of witnesses by classifying the text into categories representing the emotions the witnesses felt when they gave their testimonies. Sentiment analysis was conducted to calculate the frequency distribution for each emotion in each sentence. Then, these sentences were combined to compute the overall percentage of each emotion.

### 3.6. Ethical concerns

In line with the ethics approval process for this study, all the respondents and the agencies they worked for were anonymised. Before the interviews, the participants were again reassured of anonymity and confidentiality in their identification and responses. Another key ethical issue was informed consent and the right to privacy. All participants were assured that they were free to participate in the study and had the right to withdraw at any time without prejudice to pre-existing entitlements. Free and informed consent was achieved by having the participants sign a consent form after they had read and understood the purpose of the study. Additional safeguards were also put in place to ensure the confidentiality of the data and participants’ security. To strengthen confidentiality, every attempt was made to remove the data from a computer. The data were then transferred onto a USB stored in a locked cabinet. Table 2 outlines the procedures employed to protect participants and maintain data confidentiality.

**Table 2:** Procedures to maintain anonymity and confidentiality.

Area of Potential Risk	Specific Actions Taken to Avoid Risk
Avoiding harm to participants who can potentially be affected by the research.	• All questions were carefully selected to ensure that no harm came to the participants.
Ensuring the anonymity of all participants.	• All participants were advised before and before the study that their identity would remain anonymous and information confidential. • The names of the participants were never used in the storage of any documents.
Gaining informed consent from all participants.	• Fully completed ethics forms were completed and signed by all participants.
Avoiding Deception.	• All questions were thought through and checked to ensure none of the questions were misleading.
Data Storage and Destruction.	• All data were stored on an encrypted secure computer. • No names were used to identify the data in storage.

## 4. Analysis of findings

### 4.1. Representations of moral or immoral tax avoidance

There are a variety of different groups that have a stake in the debate over what is considered moral or immoral tax avoidance. Tax accountants, for example, are primarily concerned with compliance-related issues and ensuring that their clients pay the minimum tax required by law [52]. On the other hand, politicians are often more concerned with tax fairness and taking actions that will garner popular support [33]. Government officials tend to take a more pragmatic view, focused on the tax responsibilities of the taxpayer to comply with the letter of the law [35]. Meanwhile, tax consultants focus on tax efficiency to help their clients minimize their tax liability in a legal and ethically defensible [34,47]. This practice has for too long created an uneven playing field where companies can benefit from the goods and services provided by governments in the countries where they operate but avoid paying what would be a fair contribution through taxation. While these activities are often legal, that does not make them ethical, and they impact government revenues [73,74].

**Fig 1:**
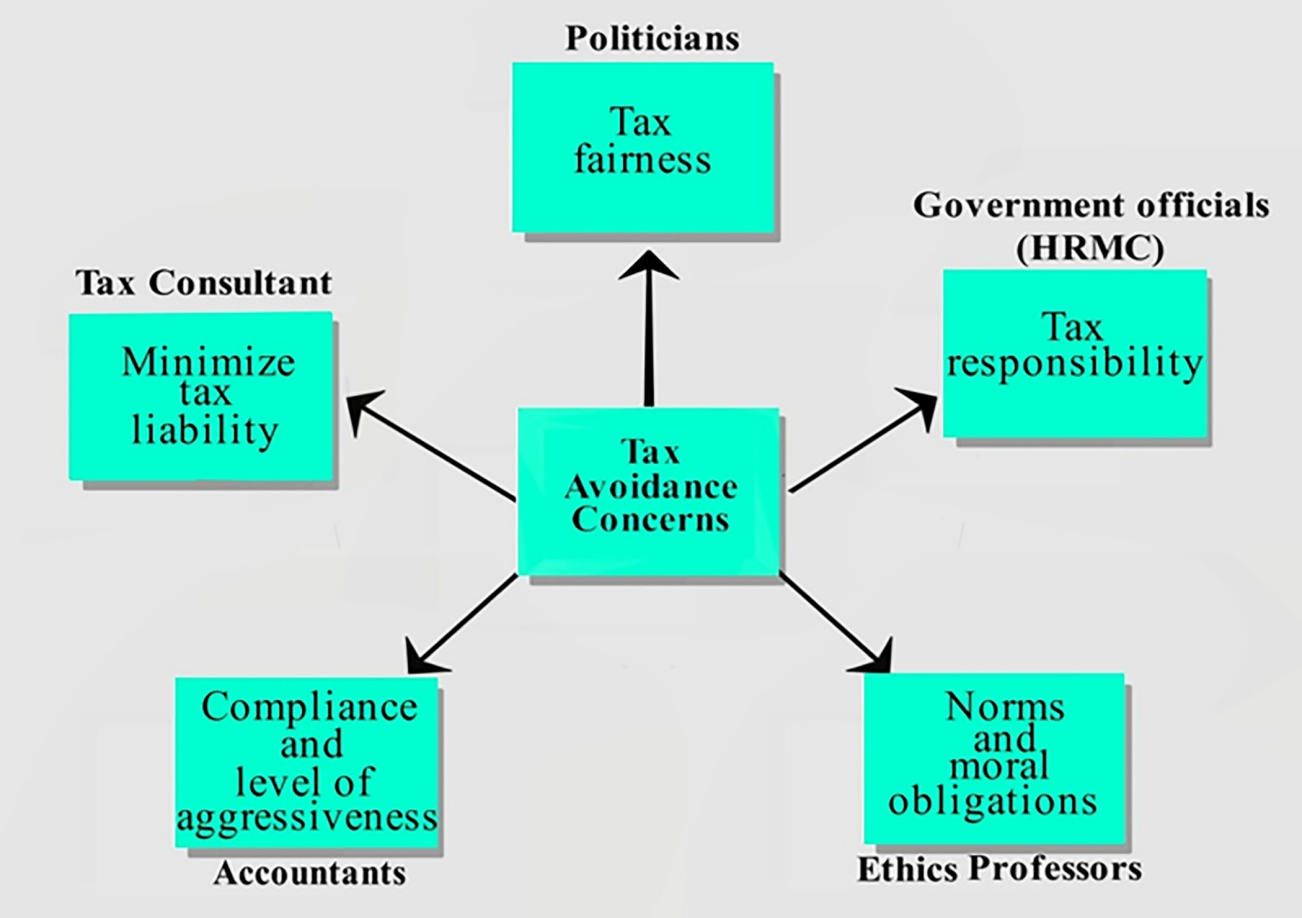
Relationship and linkages of tax avoidance.

Finally, professors tend to take a more theoretical approach, debating the abstract principles and morally suspect corporate tax avoidance practices [12,38,39]. Fig 1 shows the relationship between these stakeholders concerning tax avoidance. Each of these groups has a different perspective on what is considered moral or immoral tax avoidance, and as such, they often find themselves at odds with one another. However, it is important to remember that there is no single answer to this question—what is considered moral or immoral tax avoidance depends largely on one’s worldview and value system [35,39,75]. The following section will explore how the various groups define and understand tax avoidance.

### 4.2. Moral and immoral tax avoidance—Different perspectives

Tax avoidance within the law’s legal limits is considered a moral activity [75,76]. A senior personnel from the HMRC stated that “[t]ax avoidance is a series of generally complex accounting procedures which allows companies or individuals to pay a lower rate of income or corporation tax legally usually done so through different companies” (H1). A tax consultant also noted that “tax avoidance, unlike tax evasion, is completely legal. It simply means firms minimizing their tax liability in order to lower costs and maximize profits, and therefore tax avoidance is undertaken by the majority of firms” (TC4). An accountant with extensive industry experience noted that it is important to understand that “tax avoidance is both a legal and legitimate method of tax planning, i.e., paying the correct amount of tax through the use of current legislation, statutory instruments and the development of case law” (A5). The common theme running through these transcripts is that tax avoidance is moral and acceptable when it remains within legal limits [52].

Emerging from the discussion, however, is a discourse that some form of immoral tax avoidance is at play [38,42]. This line of inquiry differentiates between tax avoidance and tax evasion and refers to tax avoidance as a legal practice of using the letter of the tax law to minimize companies’ tax bills while still following the intent of the law and tax evasion as the illegal practice of willfully violating the tax laws to reduce one’s tax liability [33,39,51]. The accountants were particularly vocal in their resentment of tax avoidance and evasion. A senior accountant from a mid-size accounting firm reasoned that “[t]ax evasion… is illegal and it is worth pointing out that no legitimate firm would ever indulge in such practice as the consequences could extend to imprisonment” (A5). Another accountant stated that “[t]ax evasion…is illegal and should be heavily punished” (A4). Others acknowledge that “[t]ax avoidance is often viewed as a ‘dirty’ word and I believe that this is due to a lack of understanding of tax, which is actually a very tricky area of law. The morality of tax avoidance is a different argument and commercial reality prevails” (A3).

### 4.3. Gamesmanship

While there is no denying that corporate tax avoidance is a widespread problem, it is important to remember that the blame does not lie solely with the companies themselves. The army of gatekeepers who work tirelessly to find loopholes and exploit tax laws are also to blame [40,52,63,77]. These professionals are paid to find ways to reduce taxes and mitigate companies’ tax liabilities within the limits of the law [38,40,52,77]. According to a private tax consultant, the rendering of government policies to reduce tax avoidance is further circumscribed by the view that

There are too many lawyers, accountants and tax consultants, etc., whose job it is to find and exploit loopholes in the law in order to benefit their clients. Also, there will be too many countries with low business rates that companies could register in for tax purposes, even if the common EU-based ones are to change their policies. (TC2)

The well-documented use of global networks and companies to shift profits to low-tax jurisdictions is a growing problem as companies increasingly exploit the differences between tax regimes in different countries [73]. An inadequate and often compromised machinery of advisors institutionalise and facilitate the normalisation of tax avoidance to find loopholes and game the system [36,53,63]. Here, a unique dynamic of gamesmanship is at work:

I think tax avoidance is a game like everything else. Accountants and tax consultants can and will find their way for their clients to be more tax efficient, but with public pressure companies may find themselves voluntarily paying more tax. (A1)

There will always be some kind of guy who could find a way around any system you put in place. And some people are arguing well, that’s fair. That’s the game. That’s why lawyers get paid a lot of money to do these kinds of things. Um, so I think whatever we put in place, there will always be a way around it. Yeah, unfortunately. (PR2)

This naturalisation is tied to “an ever-ongoing game between law makers, accountants, and tax consultants where when one ‘loophole’ is closed accountants and tax consultants will find others” (TC1). In contemporary enterprise culture, formulating schemes to maximise corporate profits is considered to be good business acumen [41]. The accounting industry remains an important site of struggle and congealment of interest where companies can take advantage of the tax system. The logic applied here is far from passive and by no means accidental because it is accorded an intelligibility that is informed by a broader purported rationality regarding organised tax avoidance:

… you kind of have to balance the client’s needs and what is best for the client with what is ethical and what the law says. And what our regulations say as an institute. Mostly you tend to have to either, it’s a balancing act, one way or the other. You kind of have to make, it’s a very snap judgement at the time when you ask these things. You tend to have to be a bit more cautious in your initial answer to them. Otherwise, they can get a little bit more bold and you can find yourself in a situation you don’t really want to be in. (TC3)

#### 4.3.1. Moral defensiveness

The tax consultants interviewed emphasised the morality of their work and vigorously talked about advising their clients to ensure that their tax transactions did not include an illegal tax advantage. As one tax consultant put it,

If it’s an accounting issue, or they are not following an accounting policy properly, or they are not treating something properly, then we would assume that that is just because they don’t know how to do that. So, we would advise and make sure it was done properly. If it is a case when they are breaking the law, or fraud, then obviously I would speak to the partner involved, and advise him of the situation. (TC2)

A tax consultant with extensive industry experience noted that he does not operate in an ethical vacuum and that he is expected to operate in the spirt of the law:

I think there is the odd client that I have come across where things haven’t been done correctly and all that sort of thing, but we basically speak to the clients and ask them to correct things. If they don’t, then we wouldn’t act for them any longer if they were breaking the law or anything like that, if that’s what you mean. (TC4)

As the number of financial scandals continues to increase, the demand for professionals whose actions are grounded in integrity becomes increasingly critical [37]. One auditor who had been auditing publicly traded corporations for years highlighted the challenge of promoting a different way of doing business that integrated considerations and ethics:

Yes, definitely there are times when there are ethical decisions to be made… more so with the money laundering regulations that have come in because you have a personal responsibility now. It’s not just the firm, it’s actually the individuals have a responsibility to make sure that they think people are behaving properly and in accordance with the rules. So each of us individually has an obligation then to consider those aspects of the job. (A4)

This interpretation binds tax professionals to a narrow register of truth in their practices and limits their epistemic frames to technical compliance in defence of the morality of their collective actions [35,38,39].

### 4.4. Public versus private interests

The benefits of tax avoidance are considered to go to stakeholders with the strongest level of legitimacy claim [44]. The argument here is that accountants have abandoned their professional role of focusing on the public interest to focus on the commercially focused, narrow private interests of their clients:

Personally, I feel as if I do it for the client. I don’t look beyond third party except for when I am doing an audit and so forth, because obviously audit is more you are looking for fraud or misstatements, and you are looking for other people’s interests. (A4)

Not with the type of clients we deal with. They’re not sort of… we have the accounts engagement letter, or any specialist work we do always has a disclaimer in that the engagement is just between, for that purpose and not for anybody else to rely on that information. So, it’s for the clients interest I would say first. (A6)

Some participants noted that there is no denying that tax avoidance can be beneficial for shareholders. An official from the HMRC noted that “[i]t is only wealthy shareholders who are benefiting” from tax avoidance schemes (H1). Another senior HMRC official also noted that “[t]he only benefits of tax avoidance are at shareholders. Tax avoidance just makes the wealthy even wealthier. Companies can grow and prosper without the use of tax avoidance” (H2). Tax avoidance is a systematic expression of companies’ attempts to meet shareholders’ needs and remain sustainable in a competitive marketplace.

Through various interpretative frames, a different perspective on morally correct business behaviour was obtained to underline the inclusion and exclusion of stakeholders as beneficiaries of tax avoidance:

From the companies’ point of view, having less tax liabilities means more money to expand, pay shareholders and buy new assets, etc. More expansion obviously can lead to increased profits and market share. Better relations with shareholders through paying dividends as well as expanding can lead to more injection of cash and an increase in share price. (TC1)

Corporate entities are important stakeholders with a definite claim to responsible tax planning [35,44]. They are powerful stakeholders and can cease contracts if they believe that their interests are not being met. There is an innate feeling among accountants that in meeting the expectation of their clients, they are fulfilling their public service agreements: “Yeah, definitely. Especially with it being the public sector, I feel like you are kind of making sure that the taxpayer is getting value for money (A6).” Another tax consultant stated that he “believe[s] it is not possible to punish… a corporation for effectively planning its tax affairs” (TC2). Responsible tax planning is well within the limits of the law, and corporate entities cannot be faulted for having a “game plan” to mitigate their tax burden (A4). The amount of taxes that corporations should pay is evidently not a question of moral intuition [35,38]. Rather, through this sophisticated approach, individuals and organizations take advantage of opportunities created within the legal framework (e.g., IFIS) of taxation. This strategic approach can subvert both the letter and the spirit of the law, creating an unfair tax system for those unaware of or unable to utilize these techniques [54,75,77].

### 4.5. Moral concerns

As a society, we have placed great importance on the concept of paying our fair share of taxes. We view tax avoidance as morally suspect and believe that everyone should contribute their fair share to support the common good. This belief is so deeply ingrained that it often leads to valid moral concerns about tax avoidance. Margaret Hodge, the chair of the Publics Accounts Committee on Tax Avoidance in the U.K. House of Commons, problematised this issue when she questioned the head of tax at PricewaterhouseCoopers about its work outside the legal framework:

… clearly, people do not pay your fees just for you to help them fill in their tax forms. They are paying the fees to help minimise their tax bills on that spectrum of planning, avoidance, evasion. Can you tell us, for every pound in fees they pay to you—your revenue—how much, crudely, you reckon they save in tax? [78].

Other interactions were more bullish in questioning the accounting firms:

I have a question for Ernst and Young, specifically about artificiality, which was prompted by what Jane McCormick (U.K. Head of Tax, KPMG) just said. Mr Dixon, you—Ernst and Young—ran a scheme for Greene King, which you ended up losing in the courts. Basically, it involved lending £300 million to a subsidiary, and the subsidiary that received the loan offsetting the interest paid on the loan against its tax bill, but there was a series of complicated transactions that meant the loan income was not taxable. You have now lost in the courts against HMRC with this. David Milne, QC, representing HMRC, described the arrangement as “a scheme for making what would otherwise be taxable income vanish into thin air”—now that is purely artificial, is it not? [78].

These accounts contain two key features associated with tax avoidance. While any effort to legally reduce a client’s taxes is an attempt at optimization, there is also a degree of moral ambiguity associated with such strategies. This practice is one of the key functions associated with accounting firms: they are skilled at managing finances with an eye toward maximum profitability, but moral doubts are attached to their tax practices [73,75,76]. A second key complementary concern hinges against a subset of legal interpretations suggesting that taxation puts the burden on the corporation to pay taxes [35,38].

In their defence, the representatives of the accounting firms in their response to the PCBS focused on adopted a deontological approach by arguing that they were following the tax codes and that their strategies were the results of fiscally responsible and ethical tax planning. The accounting profession is not to be blamed if clients encourage fiscally responsible tax planning within the limits of the law.

You’ve got the obvious, sort of, tax avoidance ones. Because the client always wants to pay less tax. From the ethical point of view, everybody should pay what’s due. But they tend to want to either, some of them just want to use loopholes, but other ones actually can cross the line into breaking the law. (TC3)

**Fig 2:**
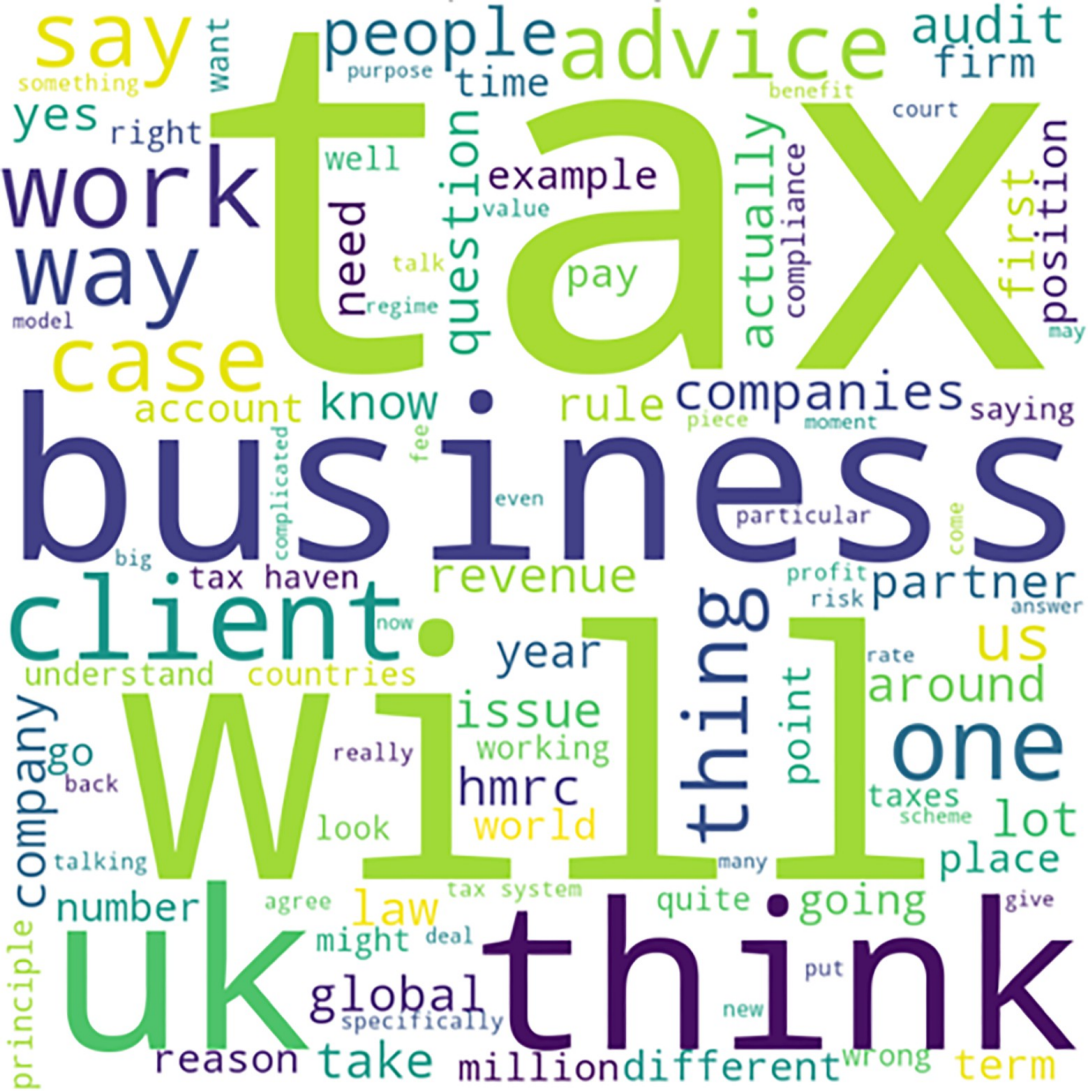
Keywords from accountants’ testimonies to the PCBS.

As can be seen from the word cloud in Fig 2, the accountants’ responses aligned with their responsibilities to provide “tax advice” to their “clients” and “businesses.” However, they also mentioned other important aspects of their job, such as “compliance,” “advice,” and “law.” These keywords suggest that tax advice and compliance are primary concerns for accountants, likely due to the fact that accounting is a highly regulated profession, and accountants must therefore ensure that they are complying with all relevant laws and regulations [38,47]. The standardized system espoused by the IFRS can enhance capital market integration globally, as international investments no longer require costly reconciliations to ensure comparability of performance measures across borders. This reconciliation helps prevent fraud and tax avoidance by providing an audit trail compliant with fair tax practices and regulations [54]. Admittedly, the accountants’ responses may have been more diffuse than those of other witnesses who testified at the PCBS hearings regarding how moral norms were applied and the extent to which they were problematized in action; however, the responses also endowed a level of regularity and calculability that encouraged arbitrage of the tax system to lower tax liabilities for corporations [35,38,43,79]. The moral dimension of responsible tax avoidance is articulated with reference to a techno-structure of professional accountants working together to ensure that their noncompliance is still within the spirit of the law [35,53].

## 5. Sentiment and emotion analysis

In recent years, corporate tax avoidance has become a hot-button issue. As multinational corporations increasingly use complex strategies to minimize their tax liability, many stakeholders are concerned whether the level of emotions and moral judgments surrounding this issue are affected by the seriousness of the tax avoidance [33,34,44]. There is no easy answer to this question. On the one hand, it could be argued that the more serious the tax avoidance, the more emotional and moral the judgment of the act [57,59]. Tax avoidance is effectively cheating the system, as those who engage in it deprive governments of significant amounts of revenue that could be used to fund public projects and services. Additionally, tax avoidance deprives the general public of access to these resources, cutting off entire populations from public goods and services such as adequate health care or education [74]. On the other hand, it could be argued that stakeholders are more likely to be emotional and moralistic about issues that directly affect them [35,44]. For example, a stakeholder group that is more affected by corporate tax avoidance will be more outraged by a company that avoids paying its fair share of taxes than stakeholders who are not directly affected. Ultimately, there is no definitive answer to this question. It is likely that different individuals representing several stakeholders’ groups will express different levels of emotional and moral judgment based on their personal experiences and beliefs [34]. To examine stakeholders’ emotional and moral judgements about corporate tax avoidance, the following sections will test the hypothesis that there is no difference in the emotional sentiments and moral judgements of different stakeholders on corporate tax avoidance.

### 5.1. Testing for differences in stakeholders’ emotional sentiments and moral judgements on corporate tax avoidance

To test Hypothesis 1, a p-value of 0.05 with a 95% confidence interval was used as the cut-off range. The actual p-value of 1.4 confirms that H_0_ was accepted and H_1_ was rejected at the 95% confidence interval. Hence, there was no statistically significant difference in the various stakeholders’ emotional sentiments and moral judgements on corporate tax avoidance. These results indicate that stakeholders’ emotions and moral judgements are not significantly different and suggests that although morality might play a role in how people feel about corporate tax avoidance, emotions are not a significant driving force. Instead, other factors such as personal preferences or political beliefs are likely to have a greater influence on stakeholders’ opinions.

Fig 4 contains the sentiment analyses of nine individuals. A closer look shows that four are high-ranked representatives from Big Four firms (Jane McCormick, UK head of tax, KPMG; Bill Dodwell, head of tax policy, Deloitte LLP; Kevin Nicholson, head of tax, PwC; and John Dixon, head of tax policy, Ernst and Young). In addition, four are political appointments (Jackson, Bacon, Swales, and Mitchell) of the Public Accounts Committee (PAC), and Amyas Morse is a representative from the National Accounting Office (NAO; see Parliamentary Commission on Banking Standards, 2013a; see also House of Commons, Committees of Public Accounts, Tax avoidance: the role of large accountancy firms, Forty-fourth Report of Session 2012–13, HC 870, April 2013). Note that the accountants’ responses in their testimonies to the PCBS on their work to decrease the amount of taxes their clients pay was accorded a form of intelligibility that was mostly positive. Rather than being revered for their work, accountants positioned themselves as experts who engaged in responsible tax avoidance that was perfectly legal and within the spirit of the law [38,42,52,76].

**Fig 3:**
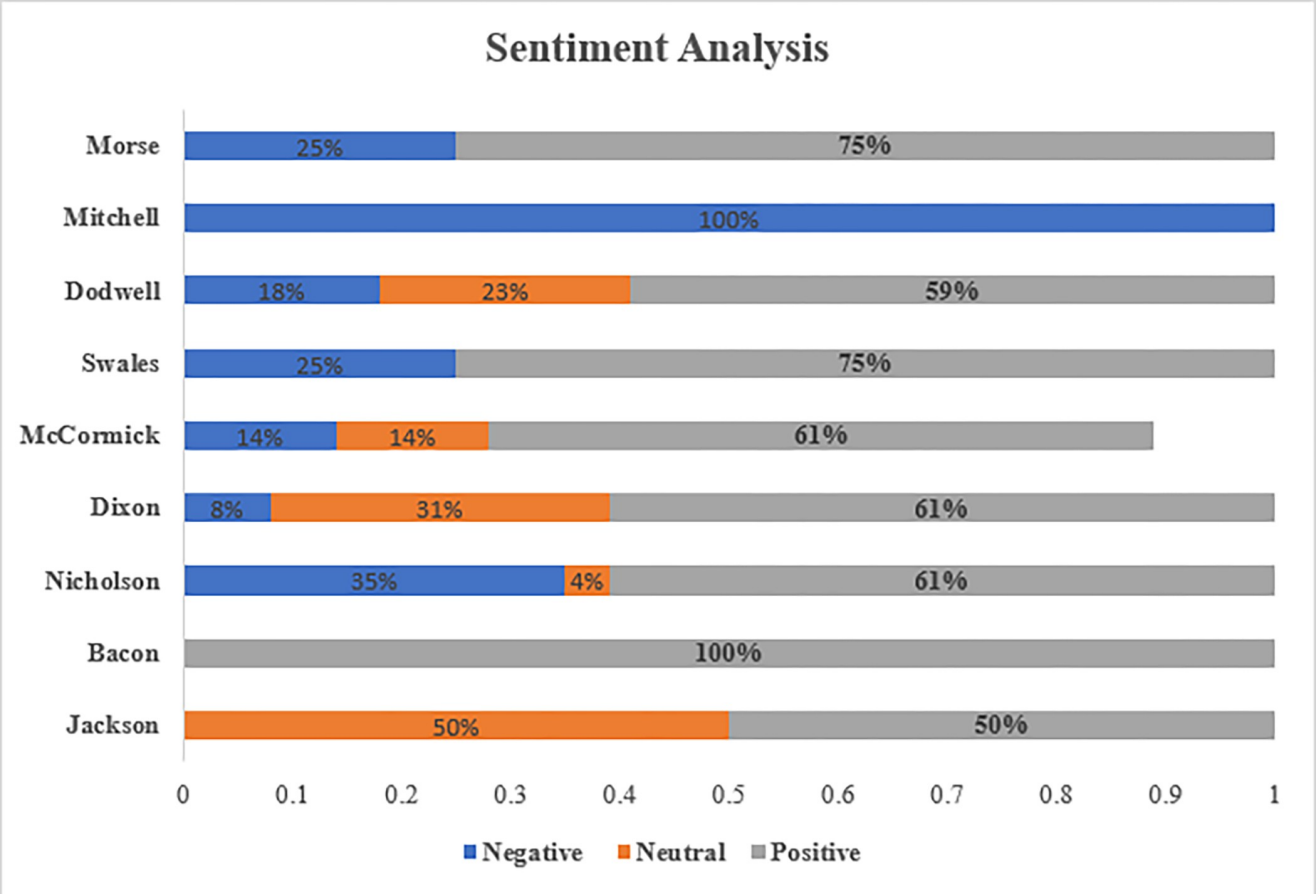
Sentiment analysis of accountants and parliamentary members response.

The results in Fig 3 provide further insight into the sentiments of PAC members and Morse on the role of large accountancy firms in tax avoidance (i.e., the accounting submissions). The findings suggested that the majority of respondents held positive sentiments; for example, Morse expressed positive sentiments 75% of the time and Jackson and Swales conveyed 50% and 75%, respectively. Bacon had a 100% positive response. However, it is worth noting that Jackson (50%), Swales (25%) and Morse (25%) also held negative views. Because Austin Mitchel is a critical PAC member, this may explain the 100% negative tone result in Fig 3. These results suggest that there is room for improvement in the way that large accountancy firms operate, but there is also general satisfaction with their work on corporate tax planning.

**Fig 4:**
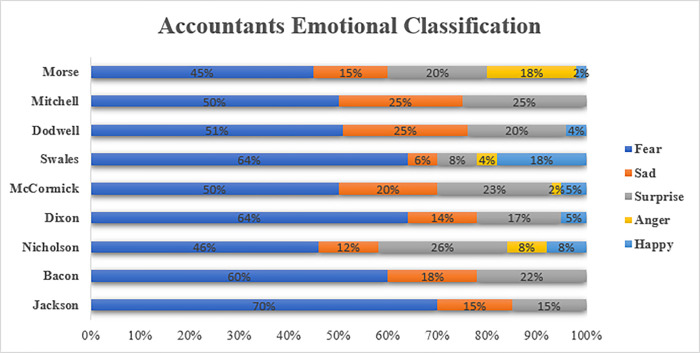
Granular sentiment analysis of accountants’ and parliamentarian response.

Even though their responses to the PCBS were positive, it can be observed from the plot in Fig 4 that for the different representatives of the accounting firms, there was a bit of *fear* and *surprise* associated with their responses to the PCBS. There was fear in the sense that the aggressive tax avoidance schemes they employed to minimise their clients’ tax burdens may be outside legal limits. In responding to a question from the chair of the PAC, Margaret Hodge, asking whether tax avoidance was a new source of profit, the accountants said that their practice remains within legal limits.

First of all, evasion is illegal and tax avoidance is not. That is the first thing we should say…All of the advice that we give … we provide under a code of conduct, which is on our website and which has been globally agreed since about 2005 or 2006. It has four or five main principles. The first is that advice has to be supportable in law—it has to be legal. When we give the advice, we have to believe that it is supportable in law. (Nicholson, [78])

Yes, I would like to say that evasion is clearly something that is illegal. It poses the possibility of criminal prosecution, and I cannot believe that anyone from any firms here or any responsible tax adviser would go anywhere near that. There is a difference in legal interpretation, which is what I think you are talking about, before the civil tribunal and court system… It is not an offence at all to have a difference of interpretation about what the law means. (Dodwell, [78])

Even though the accountants claim to be providing a legitimate service, tax reductions can be optimized depending on how the law is applied to “legally” reduce corporations tax bill [33,38]. The problem with such aggression in the application of the laws is that it borders on immorality [12]. Drawing on the accounting and finance literature, the term “aggressive” refers to an approach that aims to maximise a company’s bottom line by taking above-average risks and concentrating on high-yielding returns [43,52,53,76]. In this regard, aggressive tax avoidance represents a questionable tax position adopted by accountants and legal interpreters of the law.

Note also that all the members from the PAC and NAO expressed fear in their sentiments. Most profoundly, Jackson (about 80%) and Swales (about 75%) expressed a very high percentage of fearful sentiments. Austin (about 50%) had the lowest expression of negative sentiments. The submissions from members of the PAC and NAO suggest that there was a high level of concern about selling tax avoidance schemes that are deemed unlawful to clients (PCBS, 2013a, p. 5). As Swales argued:

You are talking about risk. You tried to define the difference between evasion and avoidance in black-and-white terms, but all of us in the room know that it is not black and white, and that is why the Chair used the expression “reasonable probability.” You are talking about risk and reputational risk, so we should not kid ourselves that this is black and white. [78]

Mr. Bacon also expressed concern by pointing out to the witnesses that

“you are saying that if something is found unlawful, as long as the intent at the start was not to evade tax, it is a civil offence and not criminal…You lost the Deutsche Bank case. That was a scheme you ran in 2003–04 to help Deutsche Bank avoid tax and national insurance contributions by using a share scheme, and £91 million of bonuses were paid through the Cayman Islands. Lots of investment banks were doing that—Goldman Sachs was the most famous. In the end, the Court of Appeal ruled against it, and most of the banks, except Goldman Sachs, shied off. At the point that the court rules against you, it shows that what you are doing is unlawful. [78]

Similarly, Mr. Mitchell opined that it

is clear that the big four use their audits—they audit more than 90% of PLCs in this country—as a base from which to sell other services … PwC put in a bid to do the audit for, I think, the Prudential, and said that it would do it for a lower price and benefit the company by providing other advice… If you are using the audit to sell services—particularly tax advice—and you sell them a scheme that they adopt, how is it legitimate for the same firm that does the audit to audit the tax scheme that they have sold to the firm? [78]

These concerns raised by the members of the PAC and NAO are not unsurprising given the potentially negative effects of corporate tax avoidance on the UK economy, and they highlight the need for government action to address this issue.

### 5.2. Testing for emotions on the moral judgements of corporate tax avoidance

Hypothesis 2 evaluates whether the level of emotions and moral judgements of the stakeholders interviewed were affected by the seriousness of the topic. The results showed that the null hypothesis was rejected, and the level of emotions and moral judgements expressed by stakeholders that were affected by the seriousness of the topic was statically significant at p < 0.05. This outcome suggests that emotions and moral judgements play a role in the sentiments expressed on corporate tax avoidance. Furthermore, the findings presented here provide valuable insights into how stakeholders perceive corporate tax avoidance and how these perceptions can be influenced by emotional and moral considerations [35,44]. There seems to be a strong negative association between corporate tax avoidance and the stakeholder’s perceptions of corporate morality that is consistent with previous research, which has shown that tax avoidance is generally seen as an immoral act [12,75,80]. Underlying these results is an implicit form of intelligibility and knowledgeability of the tax system and of the arrangements geared toward acknowledging the importance of different stakeholders in tax planning [35]. A more granular analysis of these sentiments was carried out for each stakeholder interviewed for this paper.

#### 5.2.1. Accountants

**Fig 5:**
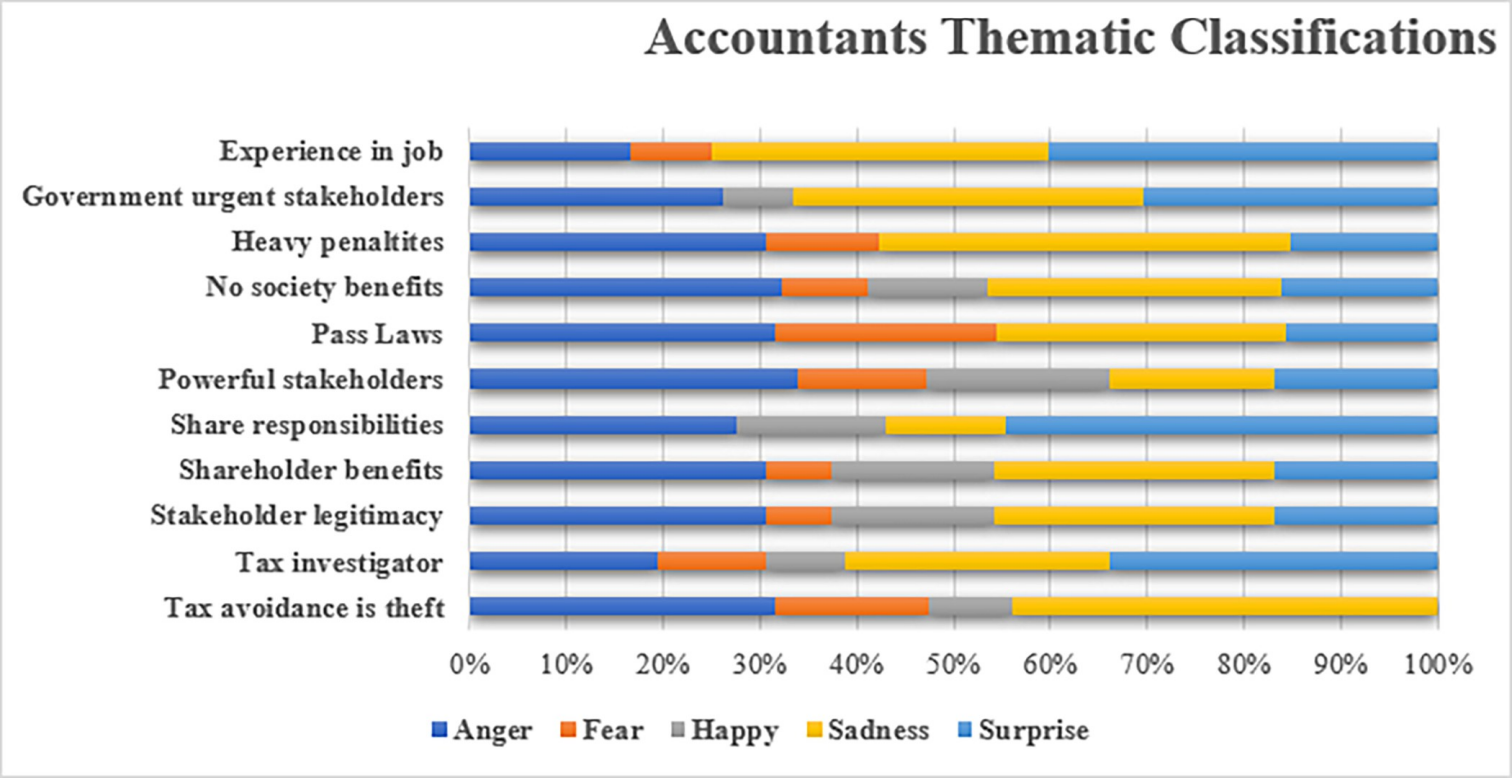
Sentiment analysis of accountants interviewed.

Professional accountants must uphold the profession’s integrity to their clients and stakeholders. This allegiance is evident in the themes of the sentiment analysis shown in Fig 5. The accountants expressed a significant number of sentiments related to issues such as “tax avoidance as theft,” their “shared responsibilities” with their clients, and their “roles” as professionals. These themes indicate that the accountants’ professional integrity is important to them, and they are committed to upholding it [12,43]. Furthermore, the significant proportion of negative emotions associated with these themes suggests that the accountants are “angry” and “surprised” about the state of the profession in dealing with tax avoidance.

This balancing act often puts accountants at the centre of heated debates over responsible tax planning. As one senior accountant articulated, the accountants’ obligations attract the full range of stakeholders with unique priorities that often lead to criticism from all sides:

Whichever way you look at it, accountants are effectively stuck in the middle in the case of tax avoidance. We are in the middle of the politics, the public outcry and the clients themselves… Accountants have a legitimate claim as if the government decides to introduce new regulations or standards in the industry then our job will be directly affected. (A1)

Another accountant who works in tax advisory noted “that tax adviser bears the name ‘Adviser’ for a reason; they are there to fill the gap where there is an absence of internal resources in a company and that companies act on their own” (A3). The accountant’s job is to ensure that their clients comply with the law and to help them minimize their tax liability within the bounds of the law. This goal is complex and challenging, but one that the professions must take seriously to maintain its professional integrity [41,52].

#### 5.2.2. HMRC

HMRC, as the UK tax authority, has been criticized for its approach to corporate tax avoidance. Large companies have avoided paying billions of pounds in taxes by using creative accounting techniques and exploiting loopholes in the law. In response, HMRC said that they have taken a hard stance against companies that attempt to minimize their tax liabilities and have pursued several high-profile cases against businesses that it alleges have engaged in aggressive tax avoidance [81].

**Fig 6:**
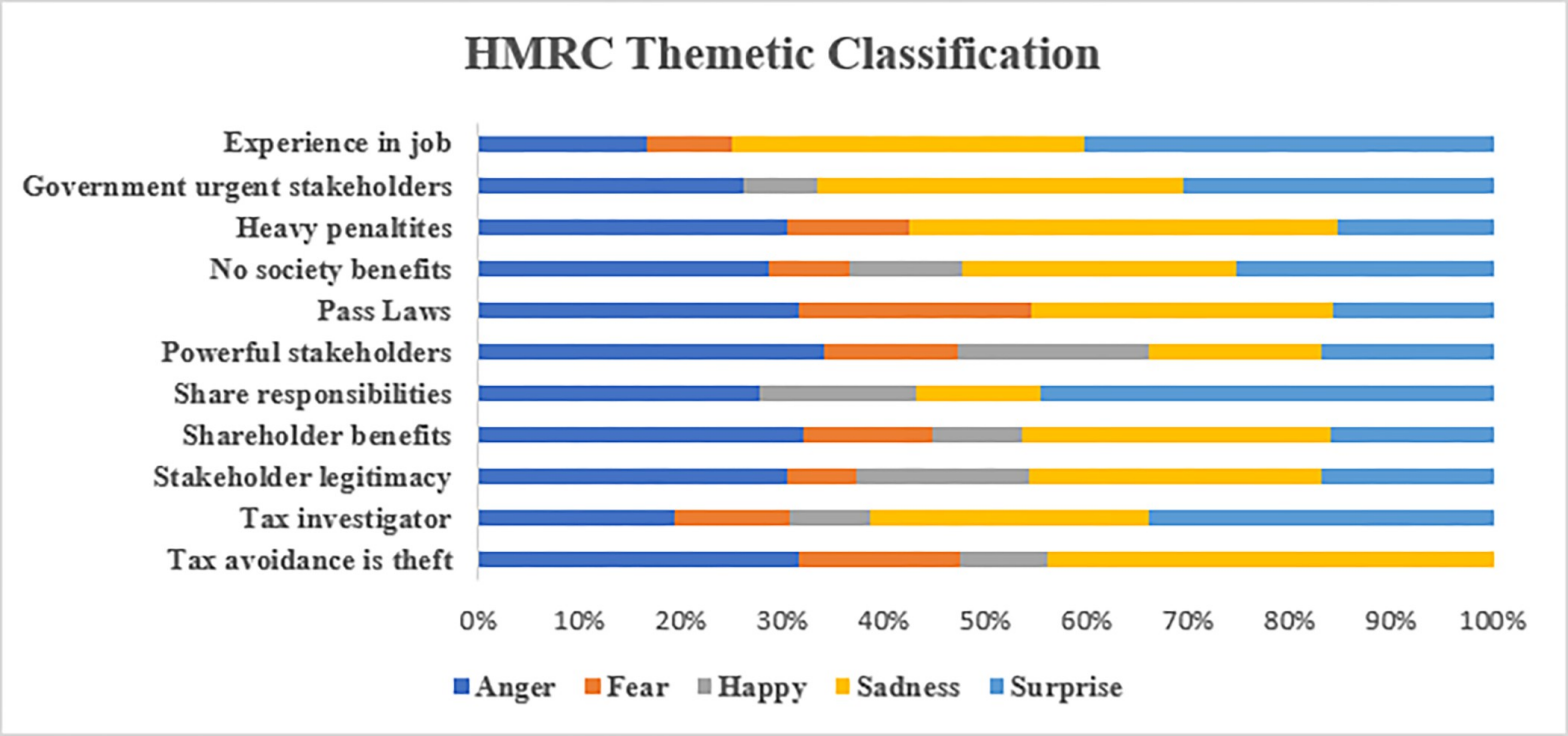
Sentiment analysis of HMRC’s participants.

Fig 6 shows the emotional sentiments of HRMC officials concerning tax avoidance and the lack of urgency in passing laws. As can be seen, the interviewees expressed emotions of “anger” about tax avoidance, asserting their belief that it amounts to theft. There is also a sense of “sadness” evident in the comments, likely due to the negative impact that tax avoidance has on U.K. public finances. However, there are also some officials who were happy with the current state of affairs, pointing to the heavy penalties that have been levied on some offenders [81]. Because the HMRC seeks to become more effective in collecting taxes, they have reasoned that one way to do so would be to implement stiffer penalties for those who do not comply. One HMRC noted “changing legislation or laws…that do not comply with the tax code is becoming a necessity” (H2). This could mean changes to legislation that would levy more financial penalties specifically for CEOs and CFOs of organizations:

I would like to see stiff financial penalties for CEO’s and CFOs of organisations. Tax avoidance is essentially theft from the public and should be punished as such. I do see it as unlikely that this will happen, as without committing a crime in the act of avoiding tax there is not much that can be done in the way of punishment. (H1)

Overall, it would appear that the HMRC’s goal is twofold: first, to ensure strict compliance through responsible corporate tax planning; and, second, to provide moral justification for their actions through a carrot-and-stick approach to secure compliance [76]. The moral justification is shifted and problematised “toward the governments as the ones who have the financial affairs of the public at heart” and that are responsible for imposing sanctions on corporations that do not comply with the tax codes (H2).

#### 5.2.3. Tax consultants

**Fig 7:**
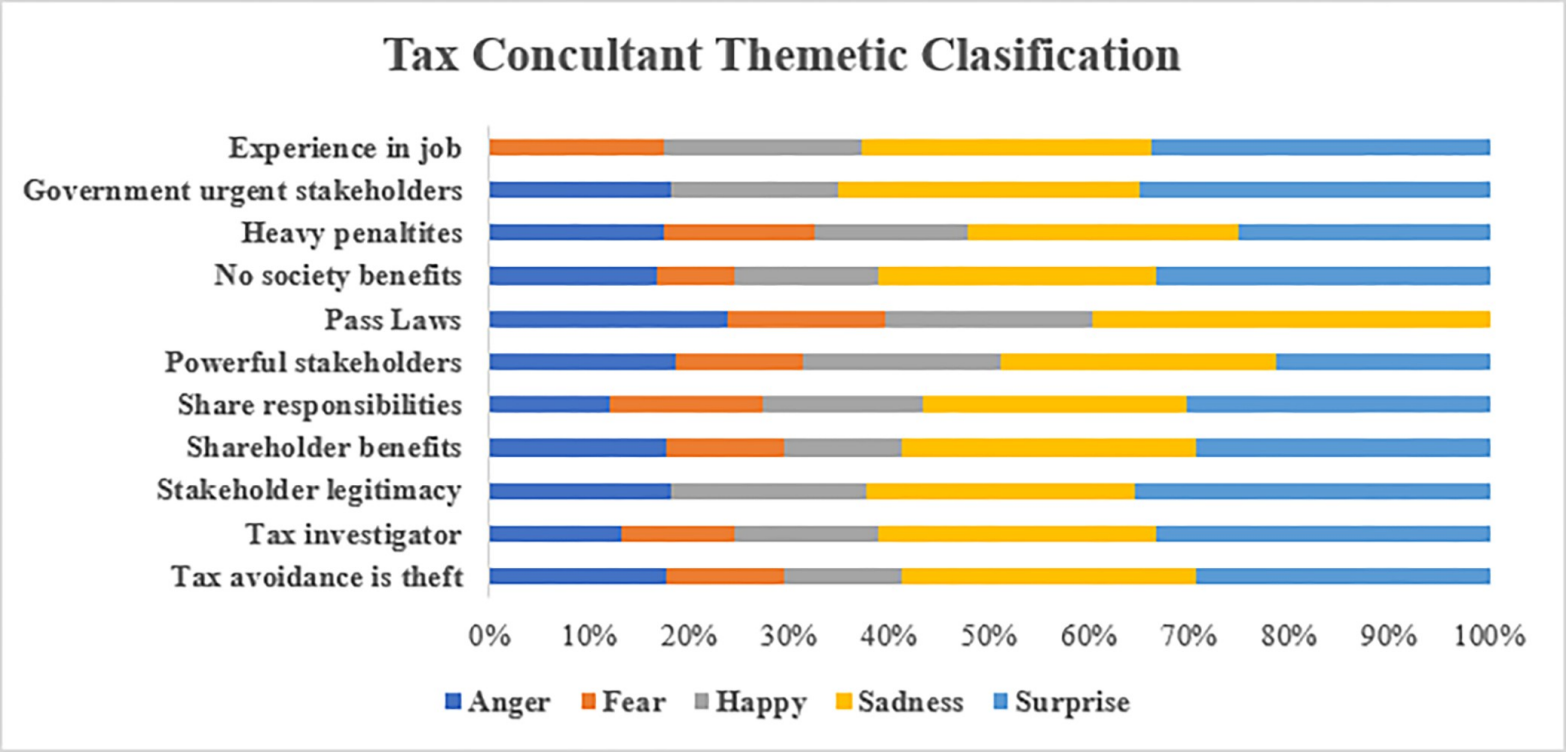
Sentiment analysis of tax consultants.

As shown in Fig 7, the tax consultants expressed emotions of “anger” about the roles of their job. There was a high percentage (25%) of “surprise” in their emotional sentiments in response to outside scrutiny involving the assertion, “tax avoidance is theft,” but no surprise when faced with the recommendation to pass more laws to scrutinize their advising roles. The fact that these professionals were feeling “anger” and “surprise” about increased scrutiny is not surprising. As one tax consultant noted “[t]ax consultants hold power as they operate in a job which often is used by companies in order to reduce their tax affairs. In other words, they have power to help companies avoid tax” (TC4). Another tax consultant with eight years of experience noted that that tax codes are complex and there are multiple ways to find loopholes to reduce the tax bill:

From what I understand it is the use of loopholes in the law that allow people or companies to pay as little tax on the money that they’re earning as possible by either having that money held in offshore bank accounts or in an area which is not under the same tax regulations as our own. (TC3)

While it may be morally wrong for companies to avoid paying their fair share of taxes, they are well within their rights to find loopholes within the limits of the law to minimize their tax liabilities [35,52]. Tax avoidance within legal limits may be seen as unfair practice; however, the fact remains that the tax code is full of loopholes and incentives that can be used to lower a company’s tax bill [41,52,53]. In fact, many experts believe that the current tax code encourages tax avoidance because it provides more opportunities for companies to minimize their taxable income [41,53]. While some argue that companies should be required to pay their fair share in taxes, the reality is that the tax codes are complex and filled with loopholes that will allow companies to take advantage and minimize their tax liability [12,33].

#### 5.2.4. Politicians

**Fig 8:**
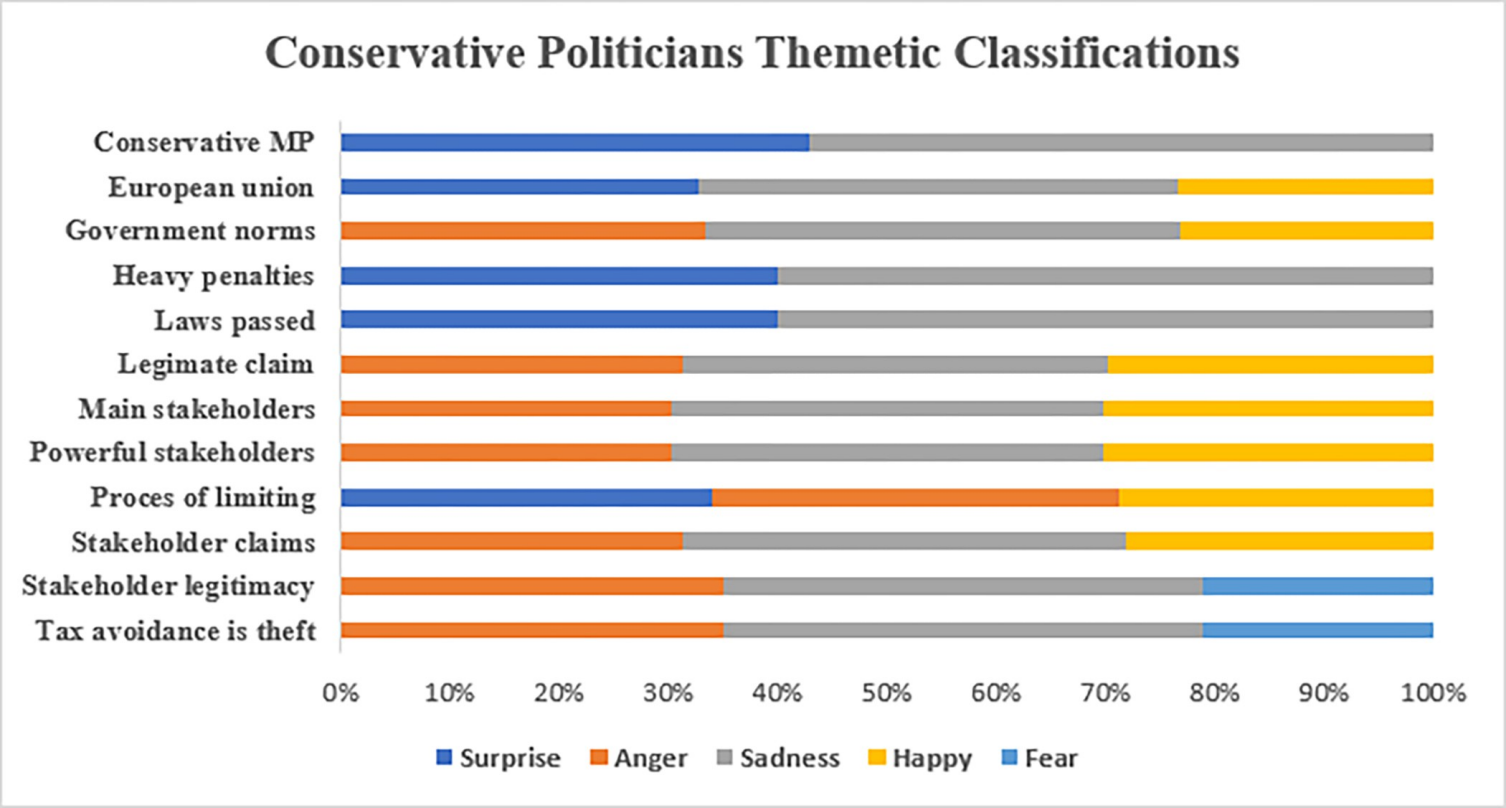
Sentiment analysis of conservative politician.

**Fig 9:**
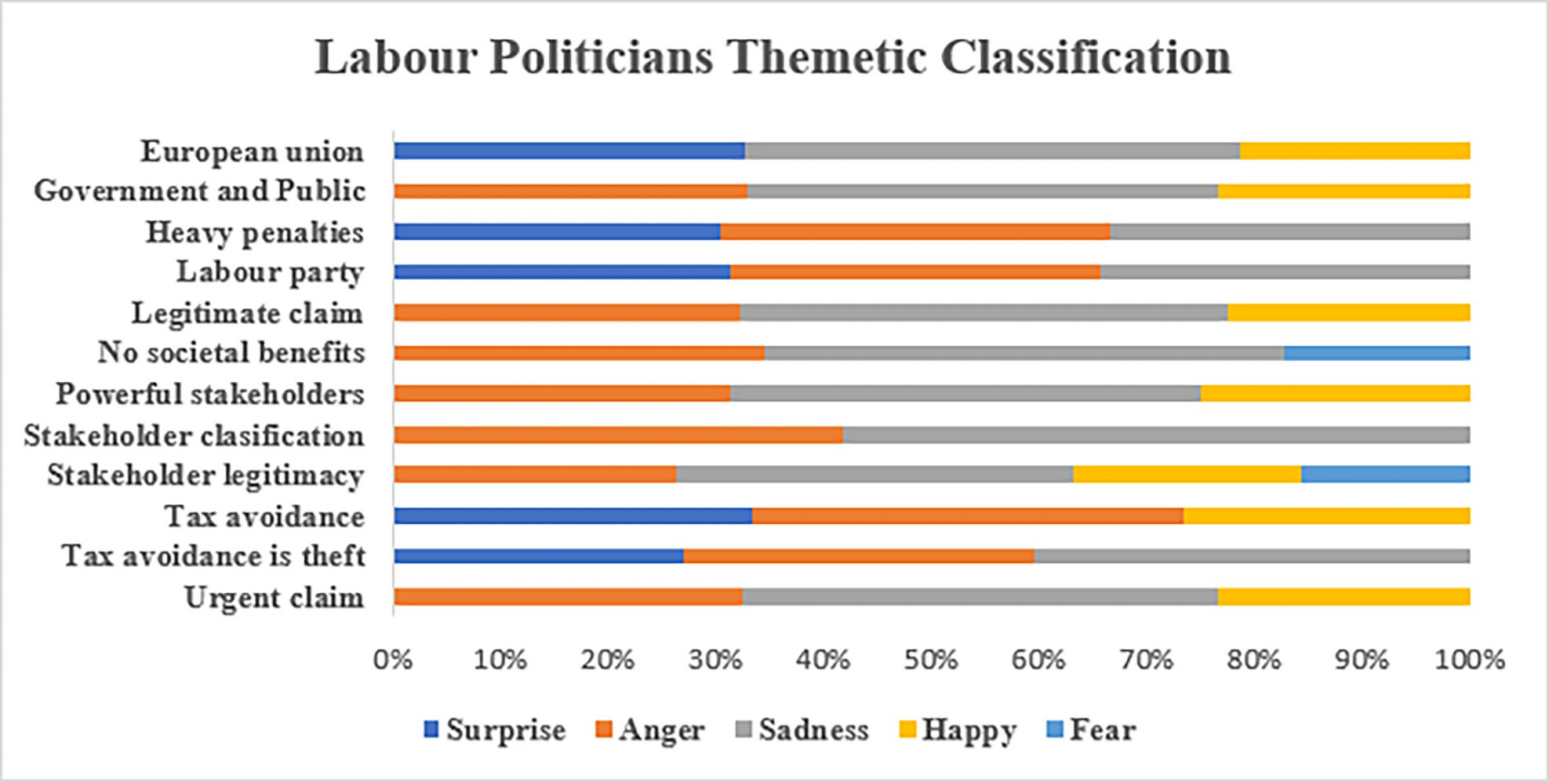
Sentiment analysis of labour politicians.

Figs 8 and 9 present the sentiment analyses of the politicians, two Labour and one Conservative. The sentiment analysis shows that both the Labour and Conservative politicians expressed sentiments of “anger” (>20%) at the theme “tax avoidance is theft.” The difference, however, is that the Labour politicians expressed more “anger” that tax avoidance is not seen as an “urgent claim,” while the Conservative politician expressed “anger” that tax avoidance not seen as more of a “legitimate claim.” In terms of penalties, all three politicians expressed “surprised” sentiments (>20%) that heavier penalties were not applied for corporate tax avoidance. Note also that both the Conservative and the Labour politicians expressed “sadness” or a sense of resignation over the overall corporate tax affairs in the U.K. These findings suggest that there is bipartisan frustration over how tax avoidance is currently perceived and dealt with by the government [35]. The need for stronger penalties could be an area of potential agreement between the Labour and Conservative parties, one that merits further exploration.

While some politicians view tax avoidance as immoral, it is important to remember that corporations are legal entities and have a moral responsibility to pay their fair share of taxes or face the legal consequences for their choices [43,48]. As one Labour politician pointed out, “stronger financial penalties” should be imposed on corporations that maintain the malicious intent to exploit loopholes in the tax code, and “custodial sentences” should be imposed for “CEOs if it can be proved they were responsible for commissioning tax avoidance” (P1). Similarly, the Conservative politician noted that he

would like to see big fines for companies who avoid tax on top of paying back what they owe to the exchequer. On top of this companies should be forced to apologise publicly for their actions. (P2)

However, it is important to remember that corporations are not morally responsible for paying taxes; they are only responsible for following the law [35,52,63]. Tax laws are complex and subject to interpretation. Corporate interests typically seek to interpret tax laws in a way that is favourable to their business, regardless of the consequences [41]. The favourable interpretation of the tax code is considered a part of the normal operational functions of companies, especially when there is competitive pressure to meet business targets [38,52,82]. This approach is often necessitated by prevailing competitive pressures that could be detrimental if not addressed [82]. As a result, these activities are an integral part of a company’s day-to-day operations, ensuring that cost and capital management can be appropriately optimized while complying with legal requirements [61]. As a result, creative tax practices, which in the past might have been characterized as fraud or tax evasion, are now viewed simply as efforts to maintain a healthy business in a hostile economic climate [61,74].

#### 5.2.5. Professors

**Fig 10:**
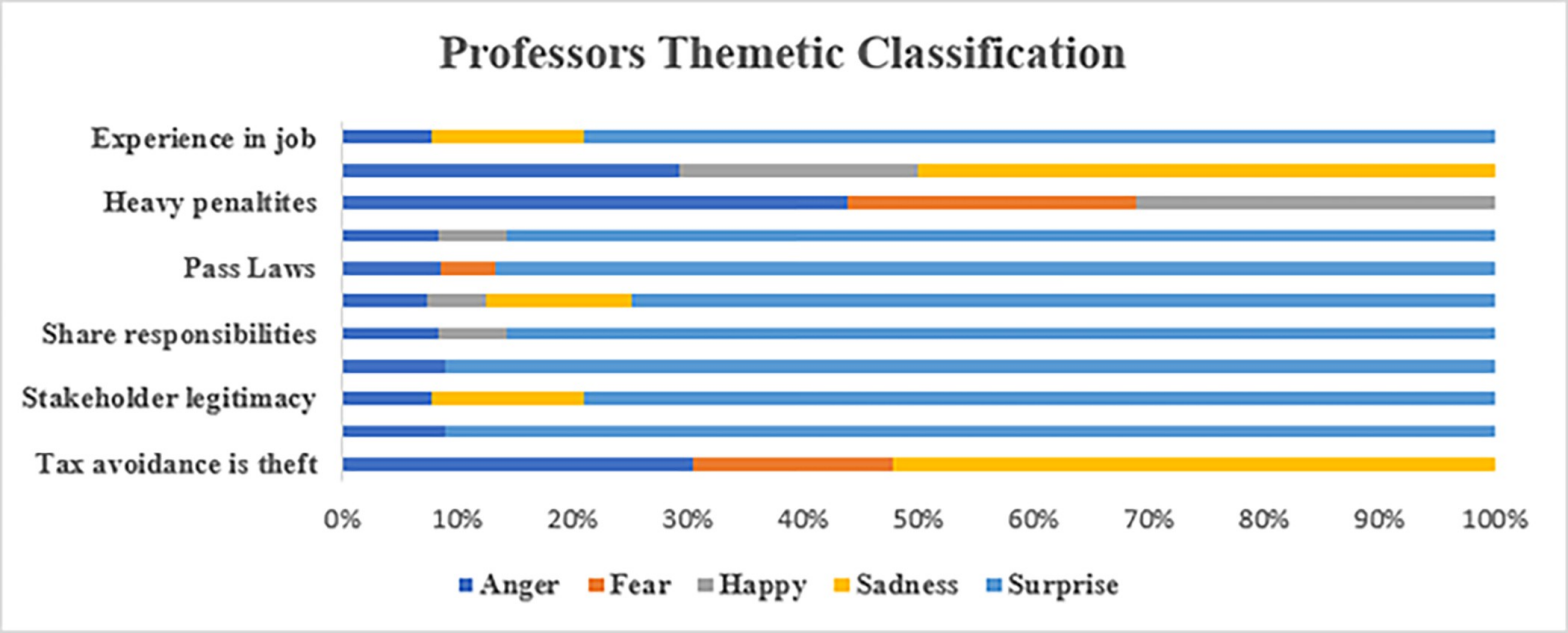
Sentiment analysis of professors.

The results of the sentiment analysis for the ethics professors interviewed are shown in Fig 10. As can be seen, the professors interviewed were generally quite “surprised” (>70%) by the extent of corporate tax avoidance. This was particularly true for themes such as the “Pass [of] Laws” and there are “no benefits to society” from corporate tax avoidance. The sentiments that “tax avoidance is theft,” and that those who engage in it should be “heavily penalized” were not met with much “surprise.” It is notable that there was also little surprise expressed concerning the idea that governments are key stakeholders of corporate tax avoidance [35]. These sentiments indicate that the government itself facilitates some form of tax avoidance. Government encourages foreign direct investment in the U.K. while providing tax breaks. Therefore, the government does not have the moral authority to condemn tax planning aimed at decreasing the corporate tax burden on moral grounds.

The morality of agents is no longer enough to mitigate corporate tax avoidance. Instead, the government needs to take a firmer stand and transpose personal integrity into the public arena via structural reforms and positive business practices that create sustainable value for both firm and industry [83]. Admittedly, some agents may be more evasive than others in terms of the manner in which moral norms are applied and the level to which they are problematized into action; however, they also endow a level of regularity and calculability that encourages arbitrage of the tax system to lower the tax liabilities for corporations [35,38,43,79]. In principle, nation states can be mobilized to exert pressure on companies to disclose their tax strategies and check for excess [40]. In this regard, corrective action to check the erosion of the tax base has been advocated as the key node of attention:

The European Union has probably the advanced collection of mutually acting states that can set something out and bring everybody else to the table. So, yes. But if Europe does things on their own, then, of course, it damages the whole of Europe. So, Europe needs to act in concert with other places (PR1).

We have this system where, um, in a globalized world, we have to have a level playing field across the whole world. So, the only reason that companies are setting up, um, their global HQ in Ireland is because Ireland has very, very favourable tax reviews. If Ireland were to decide to change that, and they are changing that law at the moment, so they aren’t going to have less favourable tax regimes, then someone like Starbucks or the Googles of the world could go to Romania, Bulgaria, they could set up anywhere else. (PR 2)

These accounts bear traces of arguments that the regulatory burden to mitigate tax avoidance lies with government legislation to deal with perceived abuse of the laws [78].

Government adoption of regulations can also result in significant tax avoidance. Therefore, administrations should pay attention to regulatory policies to prevent undue reduction of revenues for governments due to tax avoidance [84].

## 6. Conclusion

One might have expected the evidence to present a singular and unidimensional account of tax avoidance couched in the analytics of the calamitous abuse of the tax system [12,34]. Instead, what has emerged is a diversity of voices that openly reveal various lines of agreement and conflicts that a set of rudimentary assumptions and concerns regarding the nature and functionality of organised tax avoidance undergird. The tendency to circumvent laws can be attributed to beliefs that are favourable to norm-violating acts and are shaped by a greater exposure to structured capital markets that expect morally wrong actions to continue unabated [13,40,52,85]. The candour of such actions teaches some important lessons about the genesis of organised tax avoidance.

First, tax avoidance is a complex and often contentious issue, with different people understanding it differently. For some, tax avoidance is a perfectly responsible way to minimize tax liability, using legal loopholes and financial strategies [31,38,52]. For others, however, tax avoidance is seen as an aggressive and morally suspect practice characterized by corporations using their resources to unfairly minimize their tax expenses [34,41]. There is also a third category of tax avoidance which is seen as abusive, involving the deliberate misrepresentation of facts to minimize the tax bill [38,41,53]. While there is a difference between these three forms of tax avoidance, it is less clear whether there is a moral distinction between them.

Second, when it comes to moral judgments about corporate tax avoidance practices, emotions play a role for stakeholders of corporate tax avoidance. For accountants, the emotions of "anger" and "surprise" come into play on issues of responsible planning and corporate tax avoidance [43,52]. For political representatives, the emotion of "anger" is often expressed over corporate tax avoidance, as they feel that the companies are not paying their fair share. In addition, political representatives also expressed emotions of "surprise" when very little legislation has been passed to stop corporate tax avoidance. However, the political representatives saw corporate tax avoidance as a necessary evil and expressed emotions of "sadness" instead of "anger" [33]. Tax consultants and HMRC personnel are more likely to take a hard line on corporate tax avoidance if they feel it is morally wrong. However, there are growing concerns that some tax consultants are providing advice that results in corporate tax avoidance, which has led to increased scrutiny of the tax advisory industry and calls for stricter regulation [38,53]. The business ethics professors took a more nuanced view, suggesting that while there may be circumstances in which tax avoidance is morally acceptable, they noted that avoidance schemes often involve deception and dishonesty. These two qualities are generally frowned upon in corporate ethics.

The underlying message of this analysis is that the propensity of corporations to engage in tax avoidance schemes and the conditions of their existence have their roots in a constellation of rules, conditions, and logics that are themselves constitutive of structuralised capital markets [32,53,62,63]. Corporations can collaborate with tax advisors to construct intricate tax structures defined by their moral rules and accounting standards [84]. This methodology can allow these corporations to construct narrowly tailored systems to their circumstances and have a distinctive understanding of moral principles. Such complex tax schemes embed a reified sphere of activity that is carefully designed and difficult for outside entities to detect or challenge. This allows them to see and choose tax avoidance as a viable alternative to enhance their margins [38,40,52]. The choice to intentionally engage in organised tax avoidance, whether through established mechanisms or rational deliberation [85]. largely depends on the ethical culture of the corporation and on how it is decoupled from organisational practices that are geared towards profit maximisation by avoiding taxes [40].

As touched on earlier, an intricate advisory group consisting of tax advisors, lawyers, and professional accountants inform the perceived-choice process corporations use to orchestrate tax avoidance schemes. Their mastery of the schemes that is manifested *inter alia* is informed through specific practices in their advisory activities that justify tax avoidance as normal functioning in structured capital markets [40,53]. Crucially, to remain competent in the tax avoidance universe, agents of corporations have acquired in-depth knowledge of the rules and associated traits to excavate and peel back the layers of the rules and unearth the loopholes inherent in the system [13,35,52,63]. The favourable interpretations of tax laws for corporate interests, regardless of the consequences, are considered a part of the normal operational functions of companies, especially when there is competitive pressure to meet business targets [53]. In short, when people who advise businesses on legal loopholes act favourably towards norm-violating acts, it is highly unlikely that those individuals will come to view such acts as morally wrong, even when they are within the purview of the laws [52,85]. Thus, the letter of the law, evidently, is not the same as its spirit; rather, individual personal assessments of what is perceived to be morally right or mainly wrong become the centrality of the decision-making process [35,44].

These traits are described as realistic and attainable pursuits whose realisation depends on technical adjustments by tax experts to satisfy the sources of friction between being ‘habitually innovative and habitually wanting to go around regulations or to tunnel under the ring fence’ [83]. The findings of this study mirror the claims of and corroborate earlier critical business research on tax avoidance [12,48,52,53,75]. The findings reveal that the intricate details of the construction of tax schemes are ultimately a consequence of how those involved view their options (i.e., reacting to the laws governing the tax system) and subsequently make their choices (judgment or deliberation).

In the final analysis, corporations who are complicit in tax avoidance schemes are perhaps well within the limits of the law. Although this is case specific, a key point of complementarity is that such corporations may be exposed to various contexts that make them more susceptible to moral influences that encourage the construction of such schemes. These corporations are exposed to action-relevant features in structured capital markets, which are shaped and informed by contextual factors that wilfully aid and abet their propensities to engage in organised tax avoidance [13,33,34]. If legislative and regulatory adjustments do not shore up and address this example of reckless capitalism, it will be irreversible. There will be no way to escape the system or any agency left. To avoid this, tax practitioners need to be more interested in the spirit of the law and to provide advice that is morally correct rather than technically in compliance with the law [35]. Granted that tax practitioners’ ultimate goal is to minimise their clients’ tax liabilities. However, this does not exempt them from acting ethically. In seeking to maximise their clients’ margins, tax professionals and others in charge of companies’ accounting must be held to some level of responsibility for collective well-being [39].

### 6.1. Limitations and future directions

The study of tax avoidance in the U.K. is hindered by limited access to data. Not being able to analyze the problem in detail makes it difficult to accurately gauge its magnitude and how these issues are taking shape and evolving. Although some studies have tried to assess the impact of tax avoidance using available information, this study has been limited by a narrow pool of interview participants and access to secondary data to understand the magnitude and scale of the problem.

As the U.K.’s economic landscape continues to be shaped and transformed by tax avoidance, it is increasingly vital for future research to take stock of these potential limitations and understand the real implication for the economy. While understanding lost revenue is important, investigating the effect on government finances and any distributional consequences for those benefiting from public spending are also vital areas that must be explored. By thoroughly examining these matters, researchers can better understand how tax avoidance impacts individuals and groups. Consequently, acting upon these insights can help to shape official policy so that the economic outcomes of any changes crafted are far-reaching and beneficial.
